# A High-Precision Micropipette Sensor for Cellular-Level Real-Time Thermal Characterization

**DOI:** 10.3390/s110908826

**Published:** 2011-09-13

**Authors:** Ramesh Shrestha, Tae-Youl Choi, Wonseok Chang, Donsik Kim

**Affiliations:** 1 Department of Mechanical and Energy Engineering, University of North Texas, 3940 N. Elm St, Denton, TX 76207, USA; E-Mail: E-Mail: rameshstha@yahoo.com; 2 Department of Nanomechanical Systems, Korea Institute of Machinery and Materials, 156 Gajungbukno, Yuseong-gu, Daejeon 305-343, Korea; E-Mail: paul@kimm.re.kr; 3 Department of Mechanical Engineering, POSTECH, San 31 Hyoja-Dong, Nam-Gu, Pohang, Gyungbuk 790-784, Korea; E-Mail: dskim87@postech.ac.kr

**Keywords:** micropipette, thermal sensor, cellular-level, laser

## Abstract

We report herein development of a novel glass micropipette thermal sensor fabricated in a cost-effective manner, which is capable of measuring steady thermal fluctuation at spatial resolution of ∼2 μm with an accuracy of ±0.01 °C. We produced and tested various micrometer-sized sensors, ranging from 2 μm to 30 μm. The sensor comprises unleaded low-melting-point solder alloy (Sn-based) as a core metal inside a pulled borosilicate glass pipette and a thin film of nickel coating outside, creating a thermocouple junction at the tip. The sensor was calibrated using a thermally insulated calibration chamber, the temperature of which can be controlled with an accuracy of ±0.01 °C, and the thermoelectric power (Seebeck coefficient) of the sensor was recorded from 8.46 to 8.86 μV/°C. We have demonstrated the capability of measuring temperatures at a cellular level by inserting our temperature sensor into the membrane of a live retinal pigment epithelium cell subjected to a laser beam with a focal spot of 6 μm. We measured transient temperature profiles and the maximum temperatures were in the range of 38–55 ± 0.5 °C.

## 1. Introduction

Micro-level thermal sensing is useful in studies of biological activities occurring at a cellular level. For example, temperature sensing in individual cells can provide important data for thermal therapy or detection of cancer [1], metabolomic activity [2], thyroid gland disease diagnosis [3] and heat induced denaturation of DNA and proteins [4]. In addition, with increasing emphasis on micro/nano-technology, research interests have been focused on the investigation of micro/nano-structured materials, devices and their properties. Emerging need in reducing the size of electronic devices and integrated micro/nano-electro-mechanical systems (MEMS and NEMS) provides a rationale for the growing importance of the scientific research and technological advancement in micro/nano-technology. In the design of micro-electronic devices, a particular issue that needs to be addressed is the change of thermal transport properties of the components at the micro/nanoscale [5] because a thorough understanding and knowledge of thermal behavior in micro devices is critical in controlling their performance and stability.

The study of physical phenomena at the micro scale is made possible nowadays with the developments of atomic force microscopy, scanning probe microscopy and ultrafast light sources which have enabled the measurements of the thermal, mechanical, chemical, electronic, optical, and acoustic properties of materials [6]. Extensive efforts have been made for real-time temperature measurements at a microscale level (e.g., in a biological cell), where measurement of the rapid responses of thermal activities at a cell level during biological processes can provide important physiological information [7]. As an example, line thermometers [8] were fabricated to measure the rise in temperature during laser irradiation, but the spatial extent of measurement is a line rather than a point, which degrades substantially the spatial resolution. An infrared thermograph (at a resolution of 10 μm) was used for measuring temperatures during laser milling [9] and *in vitro* cell death [10]. Although temperature measurements were made by magnetic resonance imaging thermometry [11] during laser-tissue interaction, the spatial and temperature resolutions are limited to 1 mm and ∼1 °C, respectively.

Fabrication of thermocouple probes based on a glass micropipette for the measurement of thermal responses has been demonstrated [12,13]. An improved thermal sensor design was suggested in the application of the near-field scanning thermal microscope [14,15]. This measurement scheme originated from the earlier work by Fish *et al*. [12]. It was suggested that these microscale thermal sensors can provide ultrafast (sub-microsecond) response times. Another variation of a thermocouple-based sensor was tested by Watanabe *et al*. [13]. However, reliable operation in a biological cell has not been demonstrated. In addition, the fabrication technique is intricate, requiring several processes including focused-ion beam milling [13] or drawing of a micro platinum wire [12] inside a microcapillary tube. Gold and platinum were used as thermocouple materials [12,16]. These materials are expensive and generate relatively small thermo power (*i.e*., a low level of thermoelectric voltage signals).

The temperature sensor in this study can be a hand-held device and has superior spatial resolution than existing methods such as magnetic resonance temperature imaging [11] and infrared imaging [9]. The fluorescence method also can provide a means to evaluate temperature in a cell, but it has inherent temperature measurement accuracy limitations due to the dependence of temperature on pH [17,18]. Wade *et al*. reported a temperature measurement accuracy of ±1 °C using the fluorescence method [19].

In this paper, we focus on producing an accurate and reliable sensor that can be used for measuring variations of temperatures at a single-cell level during biological processes. We have calibrated the micropipette thermal sensors using a constant-temperature water bath chamber. Microscale temperature sensing was demonstrated in retinal pigment epithelium (RPE) to check the validity and usefulness of the manufactured sensors. To this end, we have used a continuous wave laser as a heating source to focus on the cell of the micropipette temperature sensor for 1.5 seconds, which will be further detailed in Section 4.

## 2. Fabrication of Micropipette Thermal Sensors

**Figure 1.**
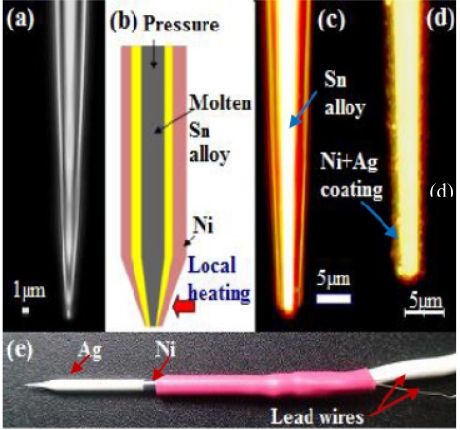
Fabrication steps of pipette thermal sensor: (**a**) an empty pipette with less than 1 μm opening; (**b**) a schematic outline of the filled pipette with a solder alloy and coated nickel; (**c**) after filling with a solder alloy and beveling; (**d**) after PVD thin film coating of nickel and silver; (**e**) a prototype micropipette thermal sensor.

The thermal sensor was made of a thick-wall borosilicate glass micropipette of the type used in various biological applications for injecting biological solutions into tissues. A borosilicate glass tube with outer diameter of 1.5 mm and inner diameter of 0.86 mm was used for producing the micropipette. The pipette puller (P-97, Sutter Instrument) was programmed according to the recipe to create patch pipettes with a tip size of 1 μm and approximately 5 to 7 mm long taper length [Figure 1(a)].

The pulled pipette was filled with a lead-free soldering alloy mainly composed of tin (Sn) by an injection molding process in conjunction with localized heating of the material. The injection molding was accomplished by mechanical pressurization (or pushing) of molten metal at the upper part of the pipette while heating the lower part near the tip with an electronic soldering gun maintained at 300 °C as shown in Figure 1(b). The next step was beveling of the tip in order to remove unwanted metal extruded outside the pipette and to sharpen the tip so that it can be injected into the cell easily. This step is particularly important to assure there is a smooth and continuous contact between two metals after physical vapor deposition (PVD) of nickel. Therefore, a BV-10 micropipette beveler (Sutter Instrument) that was designed for beveling micropipettes with tip diameters between 0.1 and 50 μm was used to sharpen and flatten the tip. A 2-axis micromanipulator consisting of an angle plate to clamp the pipette was used to adjust the bevel angle between 25 and 30°. Controlled advancement of the pipette to an ultrafine grinding surface (with 0.3 μm alumina abrasive) was performed with use of coarse and fine control knobs mounted on the manipulator. The beveling process was monitored with a high magnification lens and a charge-couple device (CCD) camera (Mightex).

In order to investigate the effect of tip size on the thermoelectric power of the sensor, the pipettes were beveled such that three tip sizes were produced: 4, 10, and 30 μm. Figure 1(c) shows an image of a pipette, which was taken with a high-magnification optical microscope (Nikon Eclipse ME600) after beveling. The pipette was cleansed with ethyl alcohol using an ultrasonic cleaner (SHARPERTEK). Then a PVD technique was used to coat thin films of nickel on the outer surface of the glass and thus form a Ni-Sn alloy junction (a bimetal junction) at the beveled tip [Figure 1(d)]. For biological applications, we coated silver (Ag) near the tip region to completely avoid the exposure of the Ni layer to a biological cell [Figure 1(e)] due to the toxicity of Ni.

To connect the sensor with a nanovoltmeter, lead wires (Sn alloy and Ni) were constructed at the end (opposite to the tip) of the micropipette by using the same materials as coated (Ni) and filled (Sn alloy); this will prevent creation of unwanted signals from additional metal junctions unless otherwise compensated electronically. Therefore the Ni-wire was wound around the surface of the coated Ni and secured with epoxy. Similarly, the same solder wire, with ample length, was soldered to the inner Sn-metal at the end of the pipette. To strengthen the connection and minimize the contact resistance between the wound Ni-wire and the thin film Ni on the glass surface, a heat shrink tube was used on top of the junction [Figure 1(e)].

## 3. Results and Discussion

### 3.1. Calibration

**Figure 2.**
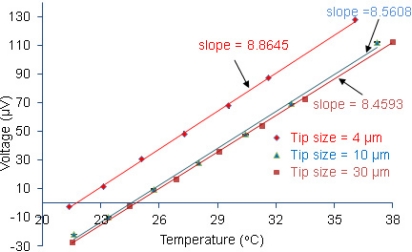
Calibration of sensors varying in tip diameters from 4 to 30 microns that were produced with the same PVD conditions. The error bars are too small to be visible. The temperature refers to the digital thermometer temperature.

Calibration of the fabricated sensors was conducted in a thermally insulated calibration chamber filled with water. The temperature of the water-filled chamber was controlled at a constant level with an accuracy of ±0.01 °C, which limits the accuracy and resolution of the produced sensors. The temperature of the chamber was varied from 25 °C to 39 °C by using an in-house temperature controller integrated with an automated data logging system with LabVIEW. A high-precision digital thermometer and the fabricated sensor were immersed into the water bath. Thus, both of them were in close proximity (∼3 mm) to indicate nearly the same temperature. The voltage generated by the sensor was recorded by a nanovoltmeter (Nano Voltmeter, Keithley 2182). During the calibration process, the cold junction (Ni-Cu and Sn-Cu junction; a Cu lead wire from the voltmeter) of the sensor was maintained at a constant temperature (e.g., 24.5 °C) which causes an offset in the voltage signal and was slightly above the room temperature so that unwanted additional thermocouple effects due to the cold junction could be removed. A plot of voltage signals provided by the sensor versus temperatures measured by a high-precision digital thermometer was obtained as shown in Figure 2. The standard deviation in the voltage measurement was less than 0.018 μV which is equivalent to a temperature rise of 0.002 °C, effectively the resolution of the temperature measurement. However, the accuracy and resolution of the temperature calibration are determined by the accuracy of the water bath temperature, which is a dominant source of uncertainty in the temperature measurement.

Figure 2 depicts a linear relation between the recorded voltage and temperature increase. The thermoelectric power of the sensor can be obtained from the slope of the line in Figure 2. Thus, fitting of the measured data with a linear trend line returned slopes (Seebeck coefficients) of 8.45, 8.56, and 8.86 μV/°C for sensors with a tip size of 30, 10, and 4 μm, respectively. The acquired data show that the thermoelectric power is not strongly dependent on the tip size. These values are far below those of bulk material, the possible cause of which can be related to the thermoelectric behavior of vapor deposited thin metal film. Hill *et al*. [20], explained the dependency of thermoelectric power of a thin film using a conduction mechanism of free electrons in crystal lattice:

(1) $$S=\frac{\pi^{2}k^{2}T}{3e\varsigma }{\left\{\frac{d\;\text{ln}\lambda (E)}{d\;\text{ln}(E)}\right\}}_{E=\varsigma }$$

where *S* is the thermoelectric power, *k* is the Boltzmann constant, *e* is the electronic charge, *T* is the temperature (K), *ζ* is the Fermi energy, *λ* is the mean free path of the conduction electron, and *E* is the electron energy. Here *S* is a function of Fermi energy and mean free path (*λ*), both of which are dependent on addition of impurities and the thickness of the film. A thickness of 80–90 nm is estimated based on the measurement of film thickness on a quarz substrate using a profilometer after deposition. At this thickness, there is a possibility of formation of a large number of broad empty channels [21] incorporating impurities and structural defects. This results in a reduction of the mean free path and an increase of the electrical resistivity, which explains the reduced Seebeck (S) coefficient in thin films.

### 3.2. Microscale Characterization of the Sensor in RPE Cells

**Figure 3.**
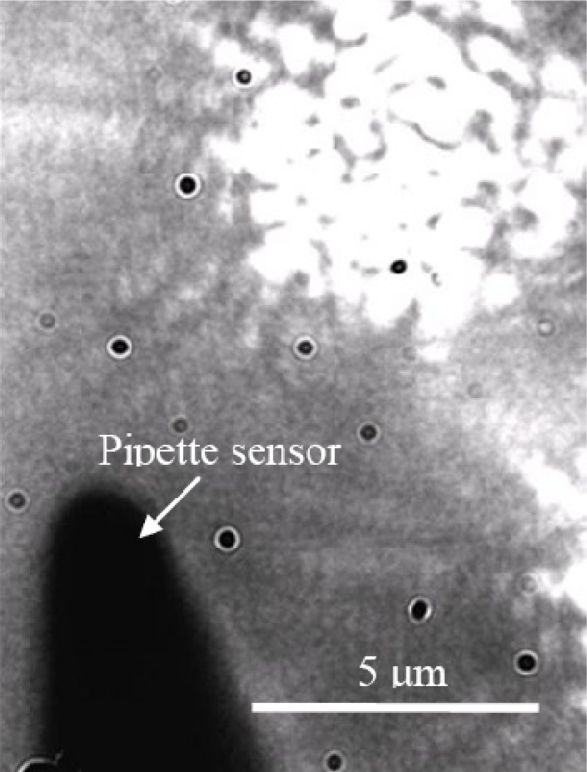
Flat-top laser beam profile with a size of about 6 μm delivered by an optical fiber and 10x objective lens.

**Figure 4.**
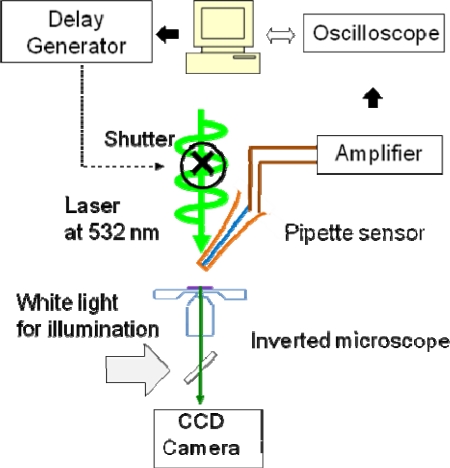
Experimental setup for the characterization and verification of f the sensor signal.

In order to show the feasibility of the fabricated sensors in biological applications, we measured transient temperature profiles induced by irradiation of a green laser beam (at 532 nm) on live RPE cells. A flat-top laser profile was created by using a 50-μm-core optical fiber and 10x objective lens. The image at the exit aperture of the fiber was delivered through the objective lens to the RPE cell with a size of 6 μm at the focal plane. The laser beam was directly irradiating the medium (not on the sensor tip) in order to avoid the damage of the tip due to the strong absorption of laser light at the tip (see Figure 3). It turned out that the amount of temperature increase by irradiating the laser with the same power but without RPE cell was around 7 °C, which signifies no damage on the sensor tip. The laser exposure time was controlled by an electro-mechanical shutter (Uniblitz, NS15B) that was connected to a delay generator (SRS DG535). The delay generator sends on-off pulses to the shutter with a time interval; in the initial test, we used 1.5 seconds of exposure time. As shown in Figure 4, generated voltage signals from the illuminated cell was amplified (SRS560) and then routed to an oscilloscope (Tektronix TDS 3054B). The transmitted data to the computer was then recorded in a text file for post data processing. It should be noted that the biochemicals on/in a cell could react with the thin silver coating. The potential reaction may in turn change the precision of temperature measurement over time. However, the control group RPE cells without laser irradiation showed a negligible tip temperature change. In addition, the size of the sensor used in this feasibility test is ∼2 μm, which is significantly smaller than the cell size (10–20 μm). Additional observation of temperatures and fluorescence (for cell viability) for 10 minutes with the sensor positioned at or inside the cell membrane shows negligible change in temperatures, which indicates that there is no significant effect of inserting the sensor into the cell membrane (e.g., mechanical stress and Ag coating) on cell temperature and cell viability.

The cell viability was confirmed by taking fluorescence images of a cell before and after laser irradiation. A calcein fluorescent dye that is well retained in the cytoplasm in live cells was used for checking cell viability. Initially existing fluorescence disappeared in some cases of strong laser irradiation, which shows cell death. The cell culture and pigmentation were performed according to the description by Denton *et al*. [22].

**Figure 5.**
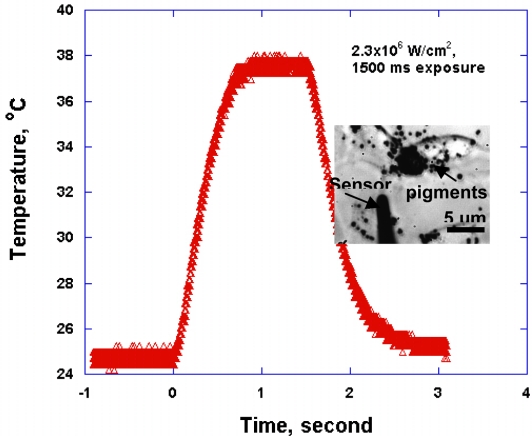
A transient temperature profile of an RPE cell irradiated for 1,500 ms. The rising time of temperature was revealed at around 600 ms. The picture inside the graph shows the RPE cell with the micropipette sensor inserted through the cell membrane.

We have measured RPE cell temperature increase during laser-cell interaction. The maximum temperatures are in the range of 38–55 ± 0.5 °C for various cells. Relatively high uncertainties in the measurement were caused by noise during transient temperature measurement. Figure 5 shows an example of a transient temperature profile of the RPE cell irradiated for 1,500 ms.

While this specific cell was alive after laser irradiation, other cells with maximum temperatures higher than 40 °C died. The rising time to reach the maximum temperature in this case turned out to be around 600 ms. The temperature plateau after 600 ms represents thermal equilibrium of the biological cell with the surrounding medium. One can thus use this sensor to find a characteristic rise time of temperature that depends on types of the surrounding media (e.g., water or cellular medium). Consequently, thermal properties of biological media, such as thermal conductivity and heat capacity, may be obtained by measuring this characteristic time (e.g., using an algorithm similar to that in [23]).

## 4. Conclusions

We have developed a novel technique for fabricating high-precision micropipette thermal sensors using inexpensive materials. The developed technique can be implemented relatively easily in a laboratory setting. The micropipette sensors were manufactured by filling a glass micropipette with Sn-based alloy and coating thin films on the outer surface of a glass micropipette by PVD so that a thermocouple junction was created only at the tip of micropipette. Sensitivity of the sensor was found to be in the range of 8.45–8.86 μV/°C, depending on the size of the sensor tips. Calibration results show that the sensor is capable of measuring a temperature fluctuation of 0.01 °C. Validity of the fabricated micropipette sensors was checked by measuring a transient temperature profile of an RPE cell exposed for 1,500 ms. The cell viability was tested with a calcein fluorescent dye. The rising time of the profile was revealed at 600 ms, which verified the sensor performance. Future work will involve use of the sensors in measuring thermal properties of biological fluids at a cellular level.
